# Inhibition of interleukin-1 receptor-associated kinase (IRAK)-4 provides partial rescue of interleukin-1 beta induced functional and gene expression changes in equine tenocytes

**DOI:** 10.1007/s11033-025-11219-2

**Published:** 2025-11-06

**Authors:** Ross Eric Beaumont, Caroline Flood, Deborah Jane Guest

**Affiliations:** Centre for Vaccinology and Regenerative Medicine, Clinical Science and Services, Royal Veterinary College, Hawkshead Lane, North Mymms, Hatfield, Herts, AL9 7TA UK

**Keywords:** Tendon, Inflammation, Horse, Interleukin-1 Receptor-Associated kinases, Interleukin-1beta, Nuclear factor kappa-light-chain-enhancer of activated B cells

## Abstract

**Background:**

Interleukin 1 beta (IL-1β) is upregulated following a tendon injury and in vitro studies have shown that it leads to numerous negative effects on tendon cell function and gene expression. IL-1β activates nuclear factor kappa-light-chain-enhancer of activated B cells (NF-κB) and we hypothesised that inhibiting NF-κB activation would mediate the negative effects of IL-1β on equine tendon cells in 3-dimensional (3D) cultures.

**Methods and results:**

Here, we tested three inhibitors of NF-κB signalling (Bortezomib, BAY11-7082 and Wedelolactone) along withTJ-M2010-5, an inhibitor of MyD88, which is a critical adaptor protein for mediating IL-1β signalling. None of these inhibitors were able to rescue gel contraction by equine tenocytes exposed to IL-1β in 3D culture. However, the daily application of the interleukin-1 receptor-associated kinase (IRAK)−4 inhibitor PF-06650833 resulted in a partial rescue of collagen contraction and interleukin-6 (IL-6) production by equine tenocytes in 3D culture. Global gene expression using RNA sequencing also revealed a partial rescue, although this was not as complete as that achieved using interleukin-1 receptor antagonist protein (IL1Ra), with many inflammatory pathways remaining upregulated. *ENPP2* expression was significantly increased by IL-1β and rescued by both IL1Ra and PF-06650833 suggesting ENPP2 may be involved in collagen contraction. However, direct ENPP2 inhibition does not rescue IL-1β mediated inhibition of contraction and ENPP2 inhibition alone reduces collagen contraction.

**Conclusions:**

Together, this data demonstrates that IL-1β has a broad mechanism of action on tendon cells which cannot be fully mediated by targeting specific parts of the signalling pathway.

**Supplementary Information:**

The online version contains supplementary material available at 10.1007/s11033-025-11219-2.

## Introduction

Tendon injuries occur commonly in humans [1] and horses [2]. To understand more about tendon injury pathogenesis and repair, in vitro studies using tendon cells (tenocytes) are widely performed. 3-dimesional (3D) systems provide a more physiologically relevant model of the native in vivo tendon environment compared to 2-dimenstional (2D) plastic culture. As the tendon is predominantly made of collagen type I, the use of a collagen matrix enables cellular binding and matrix interaction that is more similar to the native tendon than synthetic matrices [3]. 3D culture in a collagen gel can promote tendon differentiation of mesenchymal stromal cells (MSCs) [4] and embryonic stem cells (ESCs) [5]. Furthermore, we have previously demonstrated that such 3D culture allows transcriptomic differences between the tendon cells from different developmental stages to be preserved [6]. During 3D culture, tenocytes remodel the collagen gel, by aligning the collagen fibres and contracting the gel to decrease its size down to around 20% of its starting size [5, 6]. Collagen gel contraction therefore provides a useful outcome of tenocyte function and the rate of contraction can be altered in response to the growth factors and cytokines that are upregulated in the injured tendon [7–11]. Following a tendon injury there is a significant upregulation of inflammatory cytokines, with interleukin-1 beta (IL-1β) being found commonly in both horse and human tendon injury [12–14]. We have demonstrated that IL-1β has negative effects on equine tenocytes cultured in both 2D and 3D culture, eliciting changes in gene expression, inducing interleukin-6 (IL-6) production and significantly inhibiting their ability to contract a collagen gel [8, 9, 11].

Persistent inflammation is likely to contribute to the poor natural regeneration of the tendon [15] and methods to modulate inflammation have been widely investigated. Non-steroidal anti-inflammatory drugs (NSAIDs), while effective in relieving pain, may not be beneficial for tendon healing [16]. MSCs have been investigated for their anti-inflammatory effects [17–20] and have been widely used to treat horse tendon injuries [21]. Although some reports have shown positive effects in modulating IL-1β induced changes [20, 22], other studies have demonstrated little benefit [8]. Novel methods to reduce the negative impact of inflammation are therefore required.

IL-1β induces the nuclear translocation and DNA binding of the transcription factor nuclear factor kappa B (NFκB) [8, 11]. NFκB is usually composed of a heterodimer of p65 (RelA) and p50. It is constitutively located in the cytoplasm where it is bound by inhibitor of κBα (IκBα). In response to stimulation (e.g. by IL-1β or other inflammatory cytokines), IκBα is phosphorylated by IκB kinase (IKK) and subsequently undergoes proteasomal degradation. This unmasks the nuclear localisation signal from the p65/p50 dimer which gets phosphorylated and translocates to the nucleus where it can activate gene expression.

We previously tested two inhibitors of NFκB, JSH23 and IMD0354, that block NFκB translocation and inhibit IKKβ respectively [23, 24]. However, they failed to rescue IL-1β induced changes in 2D gene expression, 3D collagen gel contraction or 3D IL6 production [9]. In contrast, PF-06650833 which inhibits IRAK-4, a downstream kinase of the IL1 receptors which mediates NFκB signalling [25], modestly attenuated IL-1β induced changes in 2D gene expression, despite not rescuing 3D collagen gel contraction or 3D IL6 production when applied simultaneously with IL-1β [9].

In the present study we tested an additional three inhibitors that act on NFκB signalling: Bortezomib, a proteasome inhibitor [26], BAY11-7082 and Wedelolactone, both of which inhibit the phosphorylation of IκBα [27, 28]. We also tested TJ-M2010-5, an inhibitor of MyD88 [29], which is an adapter protein that recruits IRAK to the IL-1 receptor complex following IL-1β stimulation [30]. Finally, we determined the ability of daily application of PF-06650833 to modulate the global gene expression changes produced by IL-1β in equine tenocytes cultured in 3D and interrogated the results to identify genes which may be involved in mediating collagen contraction.

## Methods

### Tendon cell isolation and culture

Primary tendon cells were isolated post-mortem from the superficial digital flexor tendon of eight Thoroughbred or Thoroughbred-type horses (aged between 2 and 11 years. Six horses were male, two were female). Horses had been euthanised for reasons unrelated to the project and the cells were collected and used with the approval of the Royal Veterinary College Clinical Research Ethical Review Board (URN 2020 2017-2). Tendon tissue was cut into small pieces and fully digested with 1 mg/ml type 1 collagenase (Sigma-Aldrich, Dorset, UK) overnight at 37 °C, 5% CO_2_ to isolate the tendon cells as previously described [5]. Tendon cells were cultured in Dulbecco’s modified eagle medium (DMEM) supplemented with 10% fetal bovine serum, 2 mM L-glutamine, 100 U/ml penicillin and 100 µg/ml streptomycin (all Thermo Fisher Scientific, Hemel Hempstead, UK). Cells were cultured at 37 °C, 5% CO_2_ and passaged every 3–4 days with 0.25% trypsin-EDTA (Sigma-Aldrich). Cells were used between passage 4 and 7 for all experiments as we and others have previously demonstrated that although gene expression profiles can occur change between passage 0 and some very early passages, they then remain largely stable up to passage 10 [6, 31, 32]. No correlations were observed between any measured outcome and either donor age or passage number.

### 3D culture

3D culture was performed as described previously [5, 9]. 6-well plates were coated with Dow Corning Sylgard 184 Silicone elastomer (Farnell, Leeds, UK) and three pairs of 0.2 mm diameter minutien pins (Interfocus fine science tools, Cambridge, UK) were embedded 15 mm apart in each well. Tendon cells at a density of 4 × 10^5^ cells/ml were suspended in a mix of eight parts bovine collagen type 1 (PureCol; Advanced Biomatrix, Carlsbad, USA) and two parts tendon growth media with the pH adjusted to 7.2–7.6. 200 µl of the collagen-cell mix was then pipetted between each pair of minutien pins and incubated at 37 °C for 60–90 min to allow collagen setting. Tendon growth media (plus or minus IL-1β and/or inhibitors) was then added to the constructs and they were cultured at 37 °C, 5% CO_2_ for 14 days with the media replaced every 3–4 days. The constructs were imaged daily and gel width was measured using ImageJ (National Institutes of Health, USA). Gel contraction over time is shown as a percentage of the gel size at day 0 [5].

IL-1β (Peprotech, London, UK) was used at a dose of 17 ng/ml [8, 9, 11] and was added every 3–4 days during the media changes. Information on the dosage and frequency of application of each inhibitor is detailed in Table 1.

**Table 1:** The inhibitors used in this study

Name	Target	Low dose	High dose	Supplier	Administration	Dosage References
Bortezomib	26 S proteasome	20 nM	100 nM	Universal Biologicals	Every 3–4 days	[33, 34]
BAY11-7082	IKK	5 µM	15 µM	Cambridge Bioscience	Every 3–4 days	[35, 36]
Wedelolactone	IKK	1 µM	10 µM	Cambridge Bioscience	Every 3–4 days	[37]
TJ-M2010-5	MyD88	10 µM	20 µM	Cambridge Bioscience	Every 3–4 days	[29]
PF-06650833	IRAK4	100 nM	N/A	Cambridge Bioscience	Daily	[9, 38]
GLPG1690	ENNP2	1 µM	5 µM	Cambridge Bioscience	Every 3–4 days	[39]

### Interleukin 6 ELISA

IL6 was measured in the media of tendon cells cultured in 3D for 14 days under control conditions, the daily addition of 100 nM PF-06650833 alone, the addition of 17 ng/ml IL-1β alone every 3–4 days, or the combined application of 17 ng/ml IL-1β every 3–4 days and the daily addition of 100 nM PF-06650833. Media was briefly centrifuged at room temperature for 2 min 10,000 xg to remove any cell debris and stored at −70 °C until use. IL6 was measured using an equine IL6 ELISA (R&D systems, Minneapolis, US) according to the manufacturer’s instructions. Each biological replicate (five in total) was measured in duplicate on a Tecan plate reader (Infinite M Plex; Tecan, Mannedorf, Switzerland) using colorimetric detection at 450 nm with background correction at 540 nm. Sample concentrations were calculated with Assayfit Pro (AssayCloud, Netherlands) using an eight-point standard curve with four parameter logistic regression.

### RNA extraction and sequencing

Five biological replicates of equine tenocytes at passage 6 and 7 were used in RNA sequencing. These were the same biological replicates that were used in our previous study to allow the new data to be compared to our existing data using tendon cells cultured in 3D for 14 days with and without exposure to 17 ng/ml IL-1β (previous RNA sequencing data are available through NCBI GEO (https://www.ncbi.nlm.nih.gov/geo/.) under accession number GSE221370) [9]). In this study, nine collagen gels containing tenocytes that had been exposed to PF-06650833 alone, or IL-1β plus PF-06650833 for 14 days in 3D culture were harvested directly into TRI Reagent (Sigma-Aldrich) and mixed via pipetting and vortexed to completely disaggregate the gels. The RNA was isolated using a RNeasy mini kit (Qiagen, Manchester, UK). Contaminating genomic DNA was removed using a DNA-free kit (Thermo Fisher). RNA concentrations were measured using a DeNovix DS-11 Spectrophotometer (DeNovix, Wilmington, USA) and 260/280 ratios were confirmed to be ~ 2.0). RNA integrity was measured on a Tapestation (Agilent, Milton Keynes, UK) and confirmed to be >9.0. mRNA libraries and transcriptome sequencing were performed by Novogene (Cambridge, UK) on an Illumina NovaSeq 6000 to generate 31.2–41.4 million 150 bp paired-end reads per sample. Standard quality control was performed using FASTQC (version 0.11.9; Babraham Bioinformatics, Cambridge, UK). Reads did not require trimming or filtering due to the high sequence quality and minimal adapter content. Reads were aligned to the Equus caballus transcriptome obtained from the National Centre for Biotechnology Information (NCBI) EquCab3.0 GCF_ 002863925.1 using the pseudoaligner Salmon (version 1.8) [40] in Quasi-mapping-based mode with GC-bias correction. Tximport (version 1.24) [41] was used to import the quantified gene abundance data into R studio (version 4.2.1).

### Statistical analysis

The differential expression analysis of RNA sequencing data was carried out using DESEq2 (version 1.36) [42]. Genes with an adjusted p value (p-adj) of < 0.05 and a log_2_ fold change (Log_2_FC) of ± 1 were considered to be differentially expressed. Gene Ontology (GO) and KEGG pathway analysis [43] were carried out using ShinyGO (version 0.80) [44]. RNA sequencing was performed using five biological replicates which we have previously demonstrated gave us over 80% power to detect a Log2FC of ± 1 at a significance level of 5% [9].

Initial screening of the effect of inhibitors was performed on two biological replicates due to the consistent failure for any rescue effect on gel contraction and therefore statistical analysis was not performed. Likewise, the ENPP1 inhibitor was only tested on two biological replicates due to its negative effects on gel contraction and consistent failure to rescue collagen gel contraction. This data did not undergo statistical analysis.

For data that had a minimum of three biological replicates statistical analysis was performed using SPSS (version 28.0; IBM, UK, SPSS (RRID: SCR_002865)). Normality of data was checked using the Shapiro-Wilk test and the Levene’s test of homogeneity was used to determine the distribution of variances. The effect of PF-06650833 on collagen gel contraction was analysed using a two-way mixed ANOVA with Bonferroni post hoc correction for multiple comparisons. The effect of PF-06650833 on IL6 secretion was analysed using a Kruskal-Wallis test followed by a Dunn’s post hoc test with Bonferroni correction. In all cases *p* < 0.05 was considered statistically significant. These experiments were performed using five biological replicates which has over 80% power to detect a two-fold change in gel contraction (treatment versus control) at a significance level of 5% (ClinCalc.com and [8–10]).

## Results

### Inhibitors of NFκB and MyD88 do not rescue IL-1β induced changes in collagen gel contraction

**Fig. 1:**
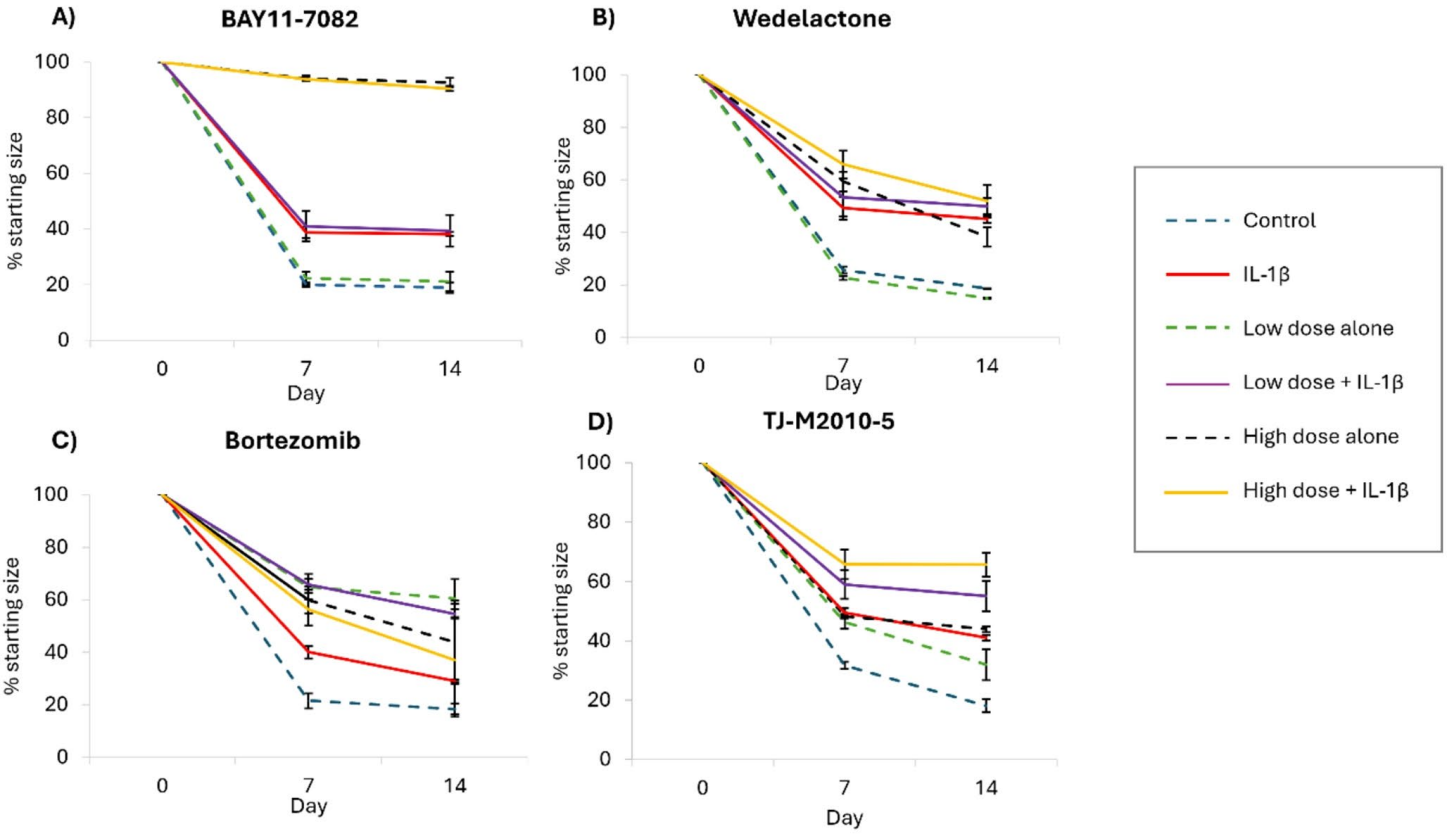
Inhibitors of NFκB (**A**, **B**, **C**) and MyD88 (**D**) do not prevent the detrimental effect of IL-1β on collagen gel contraction. Gel contraction is shown as a percentage of the starting size. Control gels (blue dash line) contract to ~ 20% of their starting size. IL-1β alone (red line) reduces contraction to ~ 40% of their starting size. Low and high doses of each inhibitor alone are shown with the green and black dashed lines respectively. The low dose plus IL-1β and the high dose plus IL-1β are shown with purple and yellow lines respectively. Error bars represent the s.e.m of two biological replicates each consisting of 2–3 collagen gels per condition

**Fig. 2:**
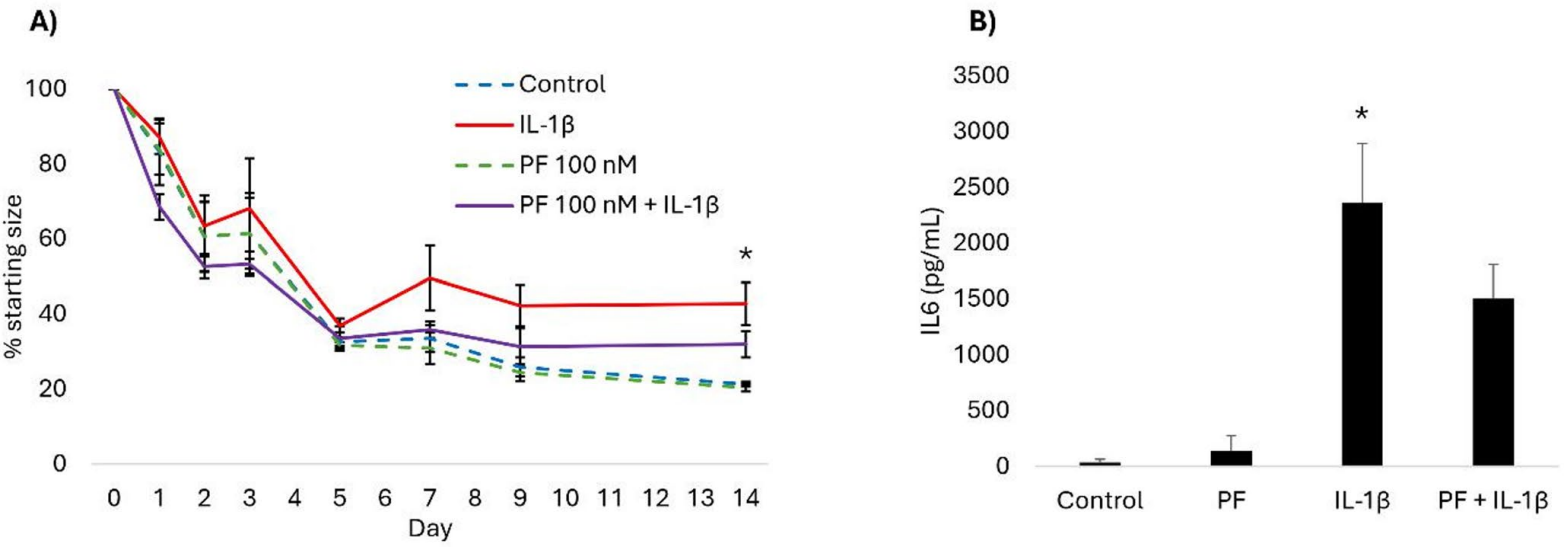
The daily application of the IRAK-4 inhibitor PF-06650833 modulates IL-1β induced changes in 3D gel contraction and IL6 production. **A** PF-06650833 alone (PF, green dashed line) has no effect on collagen gel contraction compared to the control (blue dashed line). IL-1β alone (red line) significantly impairs collagen contraction after 14 days of culture (*p < 0.05 IL-1β versus control). The combination of PF-06650833 and IL-1β (purple line) results in collagen gels which are not significantly different to the control. Error bars represent the s.e.m of five biological replicates. **B** After 14 days of culture, the 3D collagen gels produce little IL6 under control conditions, or when exposed only to PF-06650833. IL-1β results in a significant increase in IL6 production (*p < 0.05). The combination of PF-06650833 and IL-1β reduces IL6 production which is no longer significantly different to the control

As previously reported [9], IL-1β significantly inhibits collagen gel contraction over a period of 14 days of culture (Figs. 1 and 2A). In the absence of IL-1β, the collagen gels reach ~ 20% of their starting size. Whereas in the presence of IL-1β, the collage gels only reach about 40% of their starting size (Figs. 1 and 2A). Two doses of each inhibitor were tested for their ability to prevent the detrimental effects of IL-1β on collagen contraction by equine tendon cells. The NFκB inhibitor BAY11-7082 on its own at the low dose of 5 µM had no effect on collagen contraction, but did not rescue IL-1β induced changes. The high dose of BAY11-7082 (15 µM) on its own or in combination with IL-1β resulted in almost complete failure of gel contraction suggesting it was toxic to the cells (Fig. 1A). Similarly, the NFκB inhibitor Wedelolactone had no effect on collagen contraction at a low dose (1 µM) but the high dose (10 µM) alone inhibited collagen contraction and neither dose provided any rescue of the IL-1β induced changes (Fig. 1B). The proteosome inhibitor Bortezomib had negative effects on gel contraction when used on its own at both low (20 nM) and high (100 nM) doses and could not rescue the IL-1β induced changes (Fig. 1C). The MyD88 inhibitor (TJ-M2010-5) inhibited collagen contraction when used alone at low (10 µM) and high doses (20 µM) and did not show any rescue of the IL-1β induced changes.

### Daily application of the IRAK-4 inhibitor PF-06650833 enables the partial rescue of collagen gel contraction and IL6 production

We had previously demonstrated that the IRAK-4 inhibitor PF-06650833 could modestly attenuate IL-1β induced gene expression changes in 2D culture but when applied in 3D culture at the same time as the addition of IL-1β (i.e. every 3–4 days) it had no effect on IL6 production or collagen contraction [9].

Here, we determined if the daily application of PF-06650833 would enable the rescue of collagen contraction when IL-1β was continued to be applied every 3–4 days. When applied under these conditions, collagen gels exposed to a combination of PF-06650833 and IL-1β were no longer significantly different in starting size compared to control gels, although there remained a trend for them to be slightly less well contracted at day 14. Similarly, IL-1β induced IL6 production after 14 days of culture was reduced by the daily application of PF-06650833 and no longer significantly differently to the control, although the levels tended to remain higher.

### Daily application of the IRAK-4 inhibitor PF-06650833 enables the partial rescue of the global gene expression changes induced by IL-1β

**Fig. 3:**
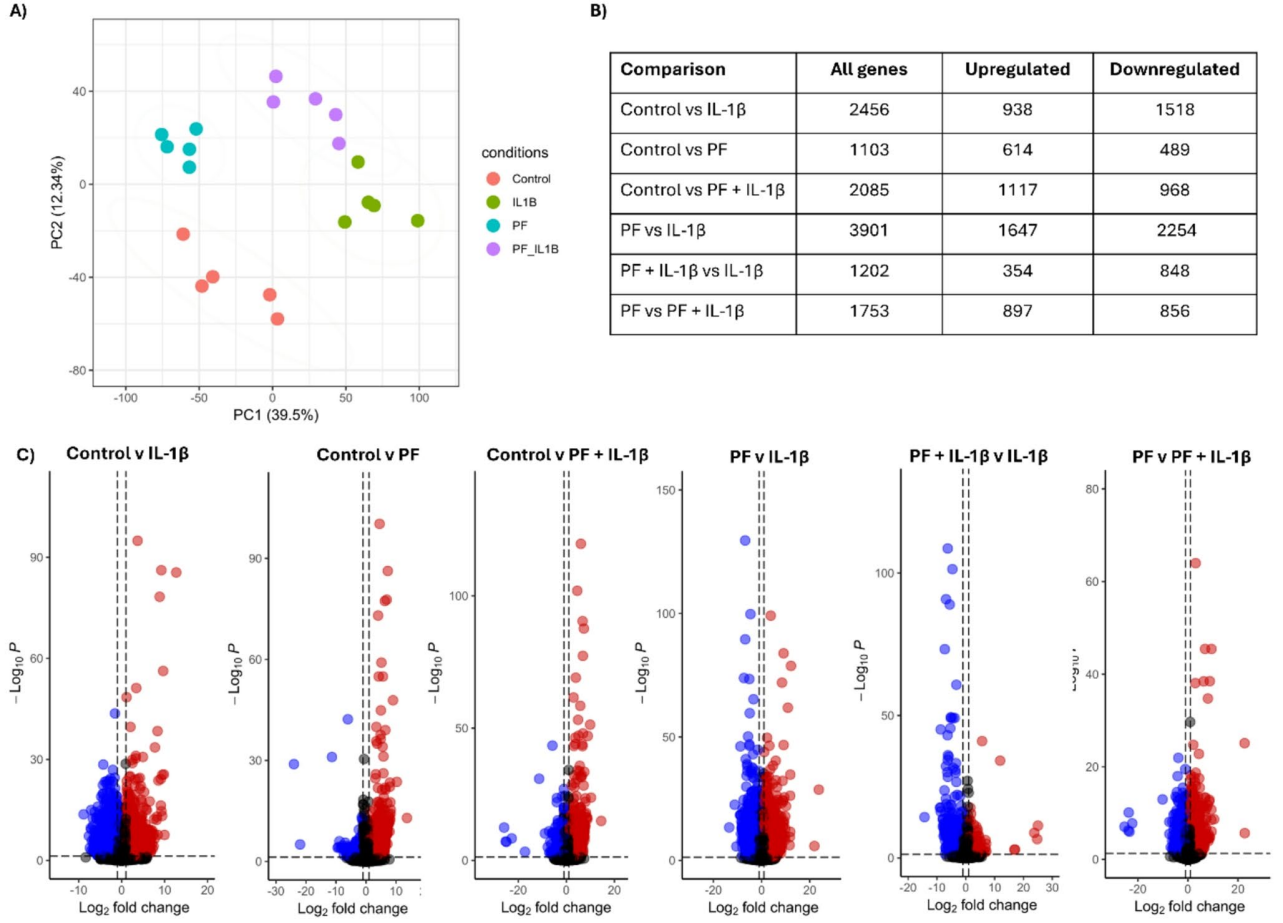
Transcriptomic responses of tenocytes cultured in 3D for 14 days in response to IL-1β and/or the IRAK-4 inhibitor PF-06650833. **A** PCA of global gene expression profiles from five biological replicates in control (red), IL-1β (green), PF-06650833 alone (blue) and IL-1β plus PF06650833 (purple). **B** Number of differentially expressed genes and their direction of change for each of the comparisons. **C** Volcano plots depicting the upregulated genes (red) and downregulated (blue) differentially expressed genes between each pairwise comparison. Differentially expressed genes had a log2FC > 1 and padj < 0.05

After 14 days 3D collagen gels containing equine tenocytes that were cultured under control conditions, IL-1β alone (added every 3–4 days), PF-06650833 alone (added daily) and IL-1β plus PF-06650833 were analysed in bulk RNA-sequencing. The control and PF-06650833 alone groups were most similar (Fig. 3A), although there were still 1103 differentially expressed genes between these two groups (Fig. 3B). The IL-1β plus PF-06650833 group, clustered in between the IL-1β alone group and the control group on PC1 which explained 39.5% of the variance (Fig. 3A). As previously demonstrated [9], IL-1β alone leads to the differential expression of 2456 genes (Fig. 3B). When comparing PF-06650833 alone to IL-1β alone there are 3901 differentially expressed genes, but this is reduced to 1202 genes when comparing PF-06650833 plus IL-1β to IL-1β alone. Only five pathways are over-represented by these genes (Supplementary Fig. 1E), but they include ECM-receptor interaction, cytokine-cytokine receptor interaction, TNF signalling subpathway and NFκB signalling pathway. The majority of these pathways are also overrepresented by genes that are differentially expressed when comparing control and IL-1β alone (Supplementary Fig. 1 A), suggesting that the expression of the genes in these pathways have not been fully rescued by PF-06650833. Similarly, the comparison of PF-06650833 alone versus PF-06650833 plus IL-1β has 1753 differentially expressed genes (Fig. 3B). Taken together this suggests while PF-06650833 alone results in some changes to global gene expression, it can partially rescue the global gene expression changes caused by IL-1β.

### Pairwise analysis of the DEGs demonstrates that many of the genes that are not rescued by PF-06650833 are involved in inflammation signalling

**Fig. 4:**
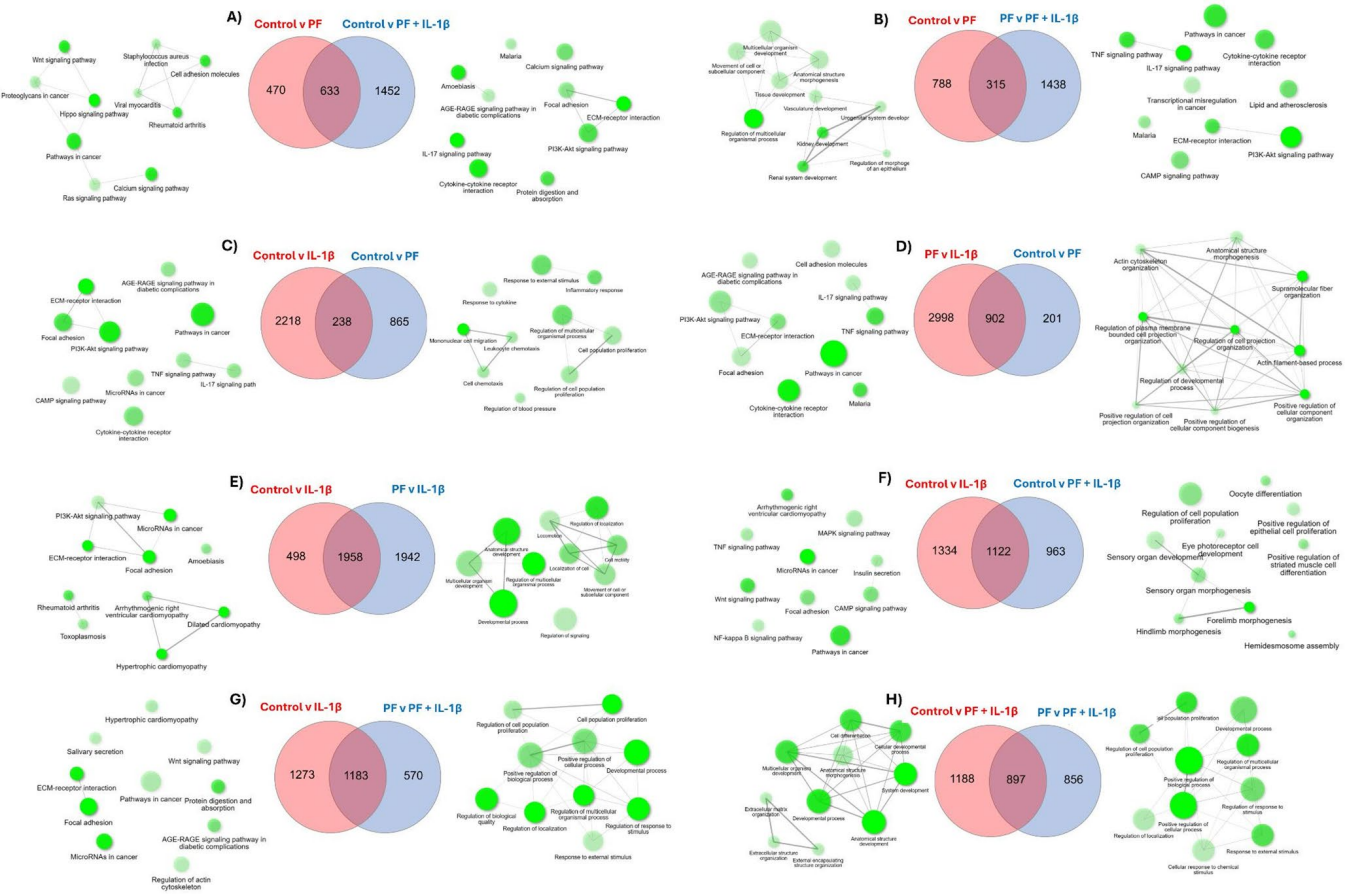
Pairwise comparisons of the differentially expressed genes. Venn diagrams showing the common and unique differentially expressed genes. Adjacent to each comparison are networks demonstrating enriched pathways over-represented by the unique differentially expressed genes. Network analysis of pathways enriched by the common genes can be found in Supplementary Fig. 2. The node shade reflects the significance of the enriched gene set, with darker nodes having a higher significance. The size of the node reflects the number of enriched genes with bigger nodes representing more genes. A thicker interconnecting line represents a stronger interaction score

Pairwise comparisons of the differentially expressed genes were then performed to compare negative control (control v PF) with groups where partial rescue was observed (control v PF + IL-1β and PF v PF + IL-1β). This was used to identify common and unique differentially expressed genes. These comparisons demonstrated that there are around 1400 genes that are differentially expressed in response to IL-1β that are not rescued by PF-06650833 and these genes are involved in pathways such as cytokine signalling and ECM-receptor interactions (Fig. 4A and B). While the majority of the genes whose expression is altered by PF-06650833 alone are unique to PF-06650833 (865 genes), 238 of them overlap with genes which are altered by IL-1β (Fig. 4C). These shared genes are overrepresented in a number of pathways, some of which have known interactions (Supplementary Fig. 2 C), but none of which are clearly linked to inflammation. In contrast, the genes whose expression changes are unique to IL-1β (> 2000 genes) and not shared with PF-06650833 are overrepresented in pathways associated with cytokine signalling, cytokine receptor interactions and ECM-receptor interactions (Fig. 4D and supplementary Fig. 2E). Of the 2456 DEGs induced by IL-1β, approximately half are rescued by PF-06650833 (Fig. 4F and G). These genes are involved in pathways which include the ECM-receptor interaction, cytokine receptor interaction, TNF signalling and NF-kappa B signalling (Supplementary Fig. 1E). However, for those genes which remain differentially expressed in the presence of IL-1β and PF-06650833, a number of inflammatory pathways are still overrepresented by the genes including cytokine signalling and receptor interaction (Supplementary Fig. 2 F and G). However, the combination of PF and IL-1β together also results in the differential expression of genes that are involved in developmental processes and structural development, proliferation and cellular responses (Fig. 4H).

### ENPP2 is involved in mediating collagen gel contraction

**Fig. 5:**
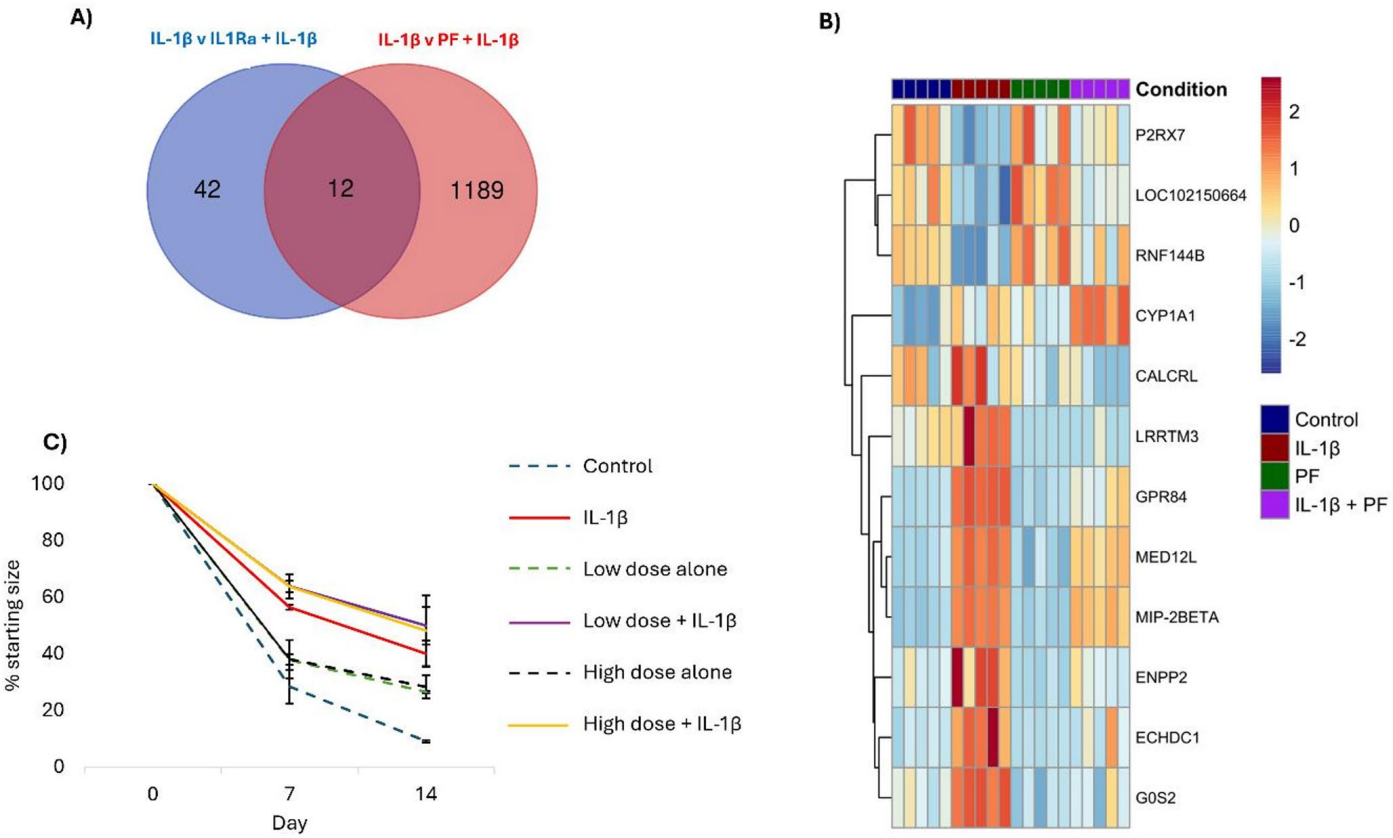
ENPP2 is involved in mediating collagen gel contraction. **A** Venn diagram showing the unique and common genes when IL-1β effects are rescued by IL1Ra and PF-06650833. **B** Heatmap depicting the expression levels of the 12 common genes when tenocytes are cultured in 3D under control conditions, exposed to IL-1β alone, PF-06650833 alone or a combination of IL-1β and PF−06650833. C) The effect of the ENPP2 inhibitor GLPG1690 on collagen contraction by tenocytes cultured in 3D for 14 days. Gel size is shown as a percentage of the starting size. Control gels (blue dash line), IL-1β alone (red line). Low and high doses of GLPG1690 alone are shown with the green and black dashed lines respectively. The low dose plus IL-1β and the high dose plus IL-1β are shown with purple and yellow lines respectively. Error bars represent the s.e.m of two biological replicates each consisting of 2–3 collagen gels per condition

We have previously demonstrated that IL1Ra can fully protect against changes in IL-1β induced changes in 3D collagen gel contraction [9] and interrogation of our RNA-sequencing data had revealed a list of potential genes that may mediate collagen gel contraction. This included nucleotide metabolism genes (*ENPP1*, *ENPP2*, *ENPP5*) and *ABI3BP*. Here we performed a pairwise analysis of the DEGs produced when comparing IL-1β vs. IL-1β + IL1Ra (54 genes) against IL-1β vs. IL-1β + PF (1202 genes). This revealed 12 common genes (Fig. 5A). Three of these genes (*P2RX7*, *LOC102150664* and *RNF144B*) are downregulated by IL-1β and their expression is at least partially restored by PF-06650833. The remaining nine genes (*CYP1A1*, *CALCRL*, *LRRTM3*, *GPR84*, *MED12L*, *MIP*-*2BETA*, *ENPP2*, *ECHDC1* and *G0S2*) are all increased in the presence of IL-1β. *CYP1A1* expression is further upregulated by a combination of IL-1β and PF-06650833, whereas for all of the other genes the IL-1β induced increases in expression are subsequently reduced by PF-06650833 (Fig. 5B). As *ENPP2* was identified in this study and our previous study, we hypothesised that blocking ENPP2 function would rescue IL-1β mediated collagen contraction. We utilised the ENPP2 inhibitor, GLPG1690, to determine the effects of ENPP2 on collagen gel contraction (Fig. 5C). However, contrary to our hypothesis both low (1 µM) and high (5 µM) doses of GLPG1690 decreased collagen contraction relative to the control, and when used in combination with IL-1β, GLPG1690 had a trend to further decrease contraction.

## Discussion

We have previously demonstrated that IL-1β has negative effects on tendon cell gene expression and collagen contraction [8, 9, 11]. These effects can be almost entirely rescued using IL1Ra which blocks the IL-1β signalling receptor [9, 11]. However, in vivo, IL1Ra has a short half-life and requires frequent administration at high doses which can lead to side effects [45, 46]. Other inflammatory cytokines, such as TNFα, are also upregulated following a tendon injury [12, 47, 48] and their negative effects are not prevented by IL1Ra [8]. As TNFα and IL-1β both activate NFκB signalling [8] we hypothesised that inhibition of NFκB could be used to block the negative effects of inflammatory cytokines. We had previously tested two NFκB inhibitors, JSH23 and IMD0354 [9]. While both were demonstrated to reduce IL-1β induced NFκB nuclear translocation, they did not rescue collagen gel contraction or gene expression changes.

In this study we tested a further three inhibitors of NFκB; Bortezomib, a proteasome inhibitor that prevents the degradation of Iκβα [26], BAY11-7082 and Wedelolactone, both of which inhibit the phosphorylation of IκBα [27, 28]. IκBα inhibits NFκB by masking its nuclear localisation signal. But the phosphorylation of IκBα leads to its degradation by the proteasome and the release of the NFκB complex which can then translocate to the nucleus to activate transcription. However, none of these inhibitors demonstrated any ability to restore collagen contraction by tendon cells cultured in 3D in the presence of IL-1β. When used alone at high doses, all three compounds themselves inhibited collagen contraction. In the case of Bortezomib, this also occurred at the low dose. As all the inhibitors act at a late stage in the IL-1β/NFκB signalling pathway, we also tested an inhibitor of MyD88, TJ-M2010-5. MyD88 acts very early in IL-1β signalling as it recruits IRAK to the IL-1 receptor complex following IL-1β stimulation which is an essential step in the downstream activation of NFκB [30]. However, the doses of TJ-M2010-5 tested in this study were not able to rescue gel contraction and in fact, TJ-M2010-5 alone inhibited gel contraction. A critical limitation of our study is that we did not perform cell viability assays, and it is possible that the inhibitors themselves are toxic to tendon cells at the high doses and the inhibition of gel contraction is due to cell death. This must be investigated in future work. Testing other inhibitors of the same pathways may also be beneficial to determine if toxic effects are drug or pathway specific. Also, in contrast to IL-1β and IL1Ra which bind to the IL1R1 receptor on the cell surface, all of the NFκB inhibitors tested here act in the cytosol, therefore their lack of efficacy in blocking the effects of IL-1β may, in part, be due to the time required to enter the cells and we did not determine if pre-treatment with the inhibitors could improve their efficacy. As the negative effects of these inhibitors was very consistent between the two biological replicates tested, we did not perform additional replicates. However, our data demonstrate that these inhibitors are unlikely to be beneficial for the treatment of tendon injuries in vivo.

We had previously demonstrated that the IRAK4 inhibitor PF-06650833 could reduce IL-1β mediated NFκB nuclear translocation, and partially rescue 2D gene expression changes and 3D collagen contraction and IL6 production, without any toxic effects on the cells when used at a concentration of 100 nM [9]. As we did not know whether IL-1β and PF-06650833 had different stabilities in cell culture, and we have demonstrated that IL-1β stimulation induces endogenous *IL1B* gene expression by tendon cells [9], we hypothesised that the daily addition of PF-06650833 during the 14 days of culture (whilst continuing to add IL-1β every 3–4 days) would lead to a greater rescue effect. Here we demonstrated that under this application regime, we no longer see significant changes in collagen gel contraction and IL6 secretion, although there is a trend that they remain different to the control conditions. This demonstrated that IRAK4 inhibition is able to partially rescue IL-1β mediated inhibition of collagen contraction.

Similarly, we demonstrated a partial rescue of the IL-1β induced changes in global gene expression. PF-06650833 alone resulted in the differential expression of 1103 genes compared to the control. With the exception of the cytokine-cytokine receptor interaction pathway, the genes were largely overrepresented in pathways linked to diseases rather than inflammation. Although there was no impact of PF-06650833 alone on collagen contraction, further work would be required to understand the impact of these gene expression changes on other aspects of tendon cell function. PF-06650833 was able to rescue around half of the genes whose expression was altered by IL-1β. These genes include those involved in NF-κ B signalling, cytokine receptor interaction and ECM-receptor interaction, which would support a positive benefit to tenocyte function as observed by a partial rescue of collagen contraction. However, approximately 1400 genes are not rescued by PF-06650833 and pathway analysis demonstrated that some of these genes are involved in the inflammatory response. Interestingly, PF-06650833 does not rescue the expression of endogenous *IL1B*, which remains significantly increased compared to the control levels (supplementary table control vs. PF_ IL1B). This suggests that PF-06650833 may not be able to fully inhibit the inflammatory response in the in vivo injured tendon. In contrast, PF-06650833 has previously been demonstrated to inhibit the inflammatory response of primary human cells in vitro at the same concentration as we utilised [38]. It has also been demonstrated to reduce autoantibody levels in a mouse model of lupus and protect against induced arthritis in rats [38]. It has also recently been tested in a clinical trial to assess its efficacy and safety in patients with rheumatoid arthritis [49]. Thus, it may have greater chance of successful clinical translation to other conditions and species. To move towards the clinical application of PF-06650833 for tendon injury future research to modify the doses of PF-06650833 and IL-1β is required. We note that, in line with our previous work [8–11, 50], we used a dose of 17 ng/ml IL-1β. However, levels of IL-1β in a surgically induced model of equine tendon injury have reported to be around 2–5 ng/ml in the tendon [13]. The levels of IL-1β produced in response to naturally occurring injury are not known. But PF-06650833 may be more effective against a lower dose of IL-1β.

As we observed a rescue in collagen contraction using PF-06650833, we hypothesised that some of the genes that were differentially expressed when comparing IL-1β alone (cells have impaired collagen contraction) to PF-06650833 plus IL-1β (cells have improved collagen contraction) mediate contraction. We compared this data to our previous data which demonstrated 54 differentially expressed genes between IL-1β alone and IL-1β plus IL1Ra (cells have normal rates of collagen contraction). Only 12 genes were shared between the two comparisons and many of them are implicated in inflammation. Three of the genes were downregulated by IL-β: *P2RX7*, *LOC102150664* and *RNF144B*. RNF11B can inhibit inflammation [51, 52] but *P2RX7* has been shown to stimulate inflammation and promote the release of inflammatory cytokines [53]. Therefore, *P2RX7* downregulation by IL-1β was unexpected. *LOC102150664* has no known orthologues at the current time. The remaining nine genes are upregulated by IL-1β. *CYP1A1* is involved in skin inflammation [54] and its inhibition enhances diabetic wound healing in a rat model [55]. However, although IL-1β mediated increases in *CYP1A1* are attenuated by IL1Ra, the addition of PF-06650833 further enhances its expression. *GPR84* [56] and *G0S2* [57] are both involved in inflammation and both IL1Ra and PF-06650833 appear to attenuate the increases in their expression observed in response to IL-1β. *MIP-2BETA* (*CXCL3*) is upregulated in diseased tendons [58] and many other inflammatory conditions [59] and its expression is also attenuated by both IL1Ra and PF-06650833. *MED12L* is a possible candidate for mediating collagen contraction as mutations lead to enhanced expression of collagen by smooth muscle cells [60]. However, the final gene *ENPP2* had also been highlighted in our previous work as possibly mediating collagen contraction [9]. ENPP2 (autotaxin, ATX) is an enzyme that generates lysophosphatidic acid (LPA), which is associated with numerous chronic inflammatory conditions [61] and contributes to fibrosis in a range of tissues [62–64]. In this study, the use of an ENPP2 inhibitor, GLPG1690 on its own inhibited collagen gel contraction. LPA has previously been demonstrated to stimulate collagen contraction by fibroblasts [65] suggesting that ENPP2 is required for collagen contraction by tendon cells. The upregulation of *ENPP2* by IL-1β is therefore unlikely to cause the reduced collagen contraction observed and may instead be a downstream effect. However, future work in this area should also measure cell viability in response to GLPG1690.

Other limitations in this study include the fact that we used donors from a range of ages (2 to 11 years) and used cells between passages four and seven. Although the expression of key tendon-associated genes has been shown to be stable across these passages [6, 31, 32], whole transcriptome profiling has not been performed and cell population doublings were not measured to more accurately reflect cell culture duration [66]. No correlations were observed between any measured outcome and either donor age or passage number, but this may reflect the small numbers of samples used.

In conclusion, we have demonstrated that IL-1β produces a range of very robust effects on tendon cells which are difficult to mediate. Blocking NFκB signalling alone fails to rescue collagen gel contraction suggesting that other signalling pathways may be activated. In contrast, inhibiting IRAK4 activity has some rescue effects but only when applied at frequent intervals. This has consequences for normal tendon gene expression and therefore may be associated with undesirable side effects if applied in vivo. The work also highlights that there is still much to be done to understand the molecular mechanisms by which tendon cells remodel a collagen matrix and bring about contraction. Increasing our understanding of this process may be essential to in the development of novel therapies to reduce the negative consequences of inflammation to allow improved tendon regeneration.

## Supplementary Information

Below is the link to the electronic supplementary material.

Supplementary Material 1

Supplementary Material 2

## Data Availability

All relevant data are within the manuscript and the supplementary information. The RNA sequencing data is available through NCBI GEO (https://www.ncbi.nlm.nih.gov/geo/) under accession numbers GSE221370 and GSE300167. The normalised count data and all differentially expressed gene lists from the different comparisons can be found in Supplementary file 2.
