# Supplementary material for: Inhibition of interleukin-1 receptor-associated kinase (IRAK)-4 provides partial rescue of interleukin-1 beta induced functional and gene expression changes in equine tenocytes
Source: Mol Biol Rep. 2025 Nov 6;53(1):54. doi: 10.1007/s11033-025-11219-2 (PMC12592280; doi:10.1007/s11033-025-11219-2)
Supplement: Supplementary file 1 — Supplementary Material 1 [file 11033_2025_11219_MOESM1_ESM.docx]

**Supplementary information**


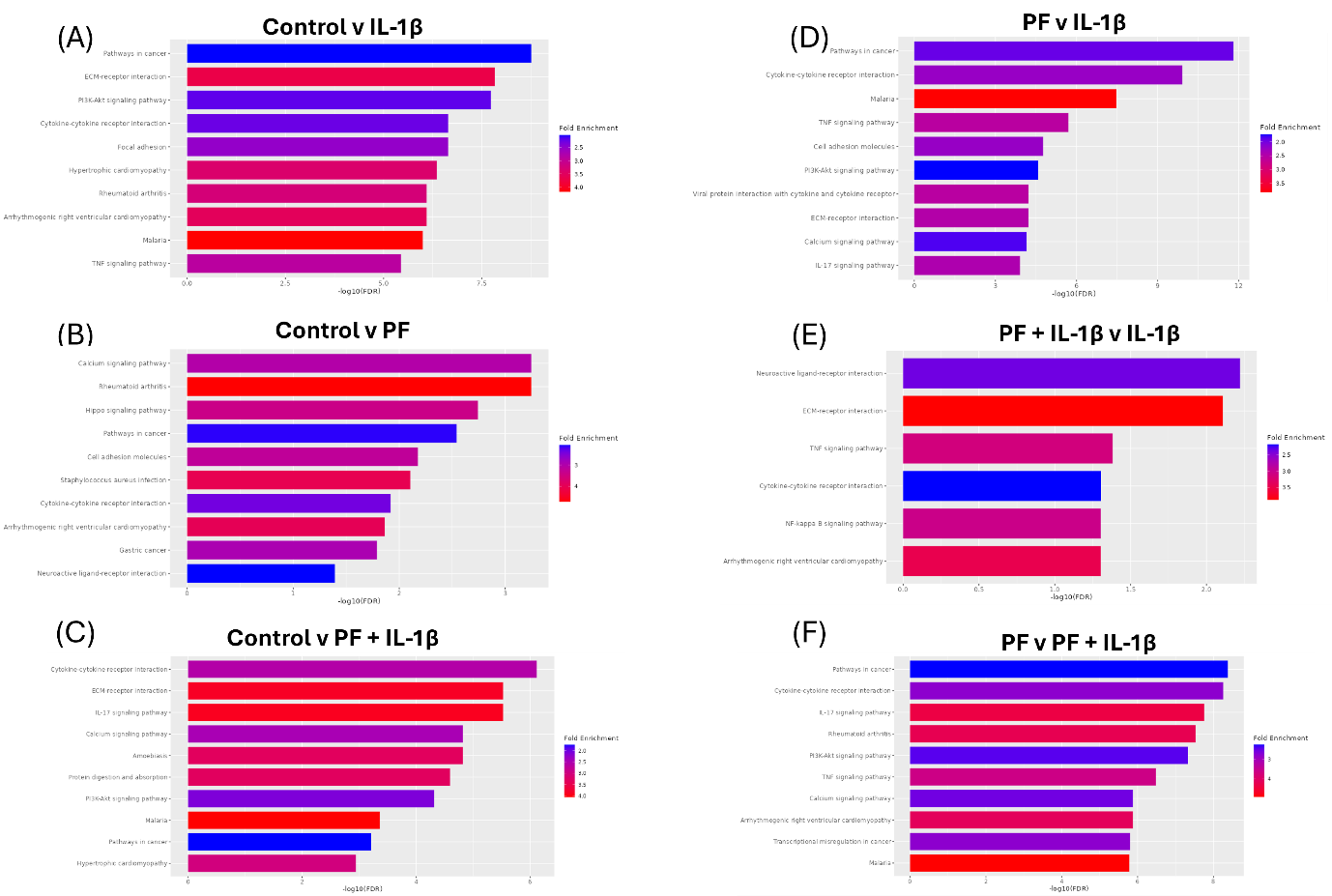


*Supplementary Figure 1. Pathway analysis of the differentially expressed genes in the different pairwise comparisons.*


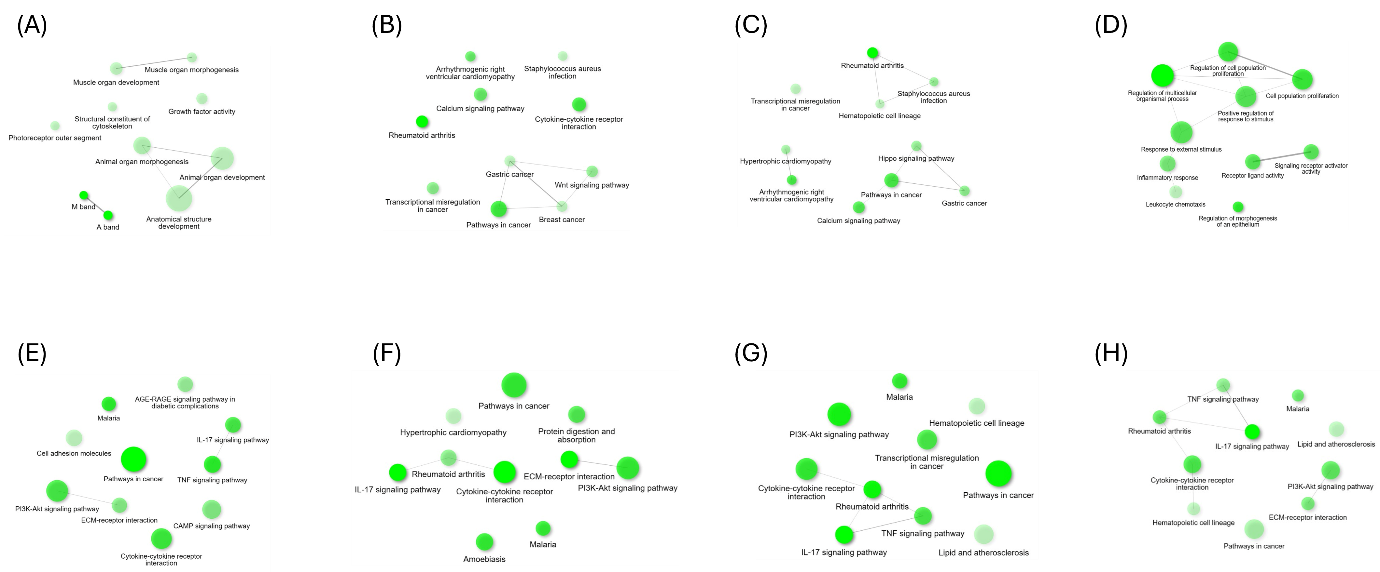


Supplementary Figure 2. Network analysis of the pathways enriched by the common genes between each pairwise analysis. A) Control v PF compared to control v PF + IL-1β. B) Control v PF compared to PF v PF + IL-1β. C) Control v IL-1β compared to control v PF. D) PF v IL-1β compared to control v PF. E) Control v IL-1β compared to PF v IL-1β. F) Control v IL-1β compared to control v PF + IL-1β. G) Control v IL-1β compared to PF v PF + IL-1β. H) Control v PF + IL-1β compared to PF v PF + IL-1β.
